# CD248 and integrin alpha-8 are candidate markers for differentiating lung fibroblast subtypes

**DOI:** 10.1186/s12890-020-1054-9

**Published:** 2020-01-21

**Authors:** Sayomi Matsushima, Yoichiro Aoshima, Taisuke Akamatsu, Yasunori Enomoto, Shiori Meguro, Isao Kosugi, Hideya Kawasaki, Tomoyuki Fujisawa, Noriyuki Enomoto, Yutaro Nakamura, Naoki Inui, Kazuhito Funai, Takafumi Suda, Toshihide Iwashita

**Affiliations:** 1 Department of Regenerative and Infectious Pathology, Hamamatsu University School of Medicine, 1-20-1 Handayama, Higashi-ku, Hamamatsu City, Shizuoka 431-3192 Japan; 2 Second Division, Department of Internal Medicine, Hamamatsu University School of Medicine, 1-20-1 Handayama, Higashi-ku, Hamamatsu City, Shizuoka 431-3192 Japan; 3 Division of Respiratory Medicine, Shizuoka General Hospital, 4-27-1 Kita Ando Aoi-ku, Shizuoka City, Shizuoka 420-8527 Japan; 4 Department of Clinical Pharmacology and Therapeutics, Hamamatsu University School of Medicine, 1-20-1 Handayama, Higashi-ku, Hamamatsu City, Shizuoka 431-3192 Japan; 5 First Department of Surgery, Hamamatsu University School of Medicine, 1-20-1 Handayama, Higashi-ku, Hamamatsu City, Shizuoka 431-3192 Japan

**Keywords:** Fibroblast, Lung, Fibrosis, Collagen fibers, Elastic fibers, Idiopathic pulmonary fibrosis

## Abstract

**Background:**

Lung fibrosis is a serious life-threatening condition whose manifestation varies according to the localization and characteristics of fibroblasts, which are considered heterogeneous. Therefore, to better understand the pathology and improve diagnosis and treatment of this disease, it is necessary to elucidate the nature of this heterogeneity and identify markers for the accurate classification of human lung fibroblast subtypes.

**Methods:**

We characterized distinct mouse lung fibroblast subpopulations isolated by fluorescence-activated cell sorting (FACS) and performed microarray analysis to identify molecular markers that could be useful for human lung fibroblast classification. Based on the expression of these markers, we evaluated the fibroblast-like cell subtype localization in normal human lung samples and lung samples from patients with idiopathic pulmonary fibrosis (IPF).

**Results:**

Mouse lung fibroblasts were classified into Sca-1^high^ fibroblasts and Sca-1^low^ fibroblasts by in vitro biological analyses. Through microarray analysis, we demonstrated CD248 and integrin alpha-8 (ITGA8) as cell surface markers for Sca-1^high^ fibroblasts and Sca-1^low^ fibroblasts, respectively. In mouse lungs, Sca-1^high^ fibroblasts and Sca-1^low^ fibroblasts were localized in the collagen fiber-rich connective tissue and elastic fiber-rich connective tissue, respectively. In normal human lungs and IPF lungs, two corresponding major fibroblast-like cell subtypes were identified: CD248^high^ITGA8^low^ fibroblast-like cells and CD248^low^ITGA8^high^ fibroblast-like cells, localized in the collagen fiber-rich connective tissue and in the elastic fiber-rich connective tissue, respectively.

**Conclusion:**

CD248^high^ITGA8^low^ fibroblast-like cells and CD248^low^ITGA8^high^ fibroblast-like cells were localized in an almost exclusive manner in human lung specimens. This human lung fibroblast classification using two cell surface markers may be helpful for further detailed investigations of the functions of lung fibroblast subtypes, which can provide new insights into lung development and the pathological processes underlying fibrotic lung diseases.

## Background

Fibroblasts play crucial roles in the wound-healing process by producing an extracellular matrix, which comprises collagen and elastic fibers, for appropriate tissue remodeling [1]. Fibroblasts show various localization patterns, even within a single organ, and each of these populations may show different characteristics [2]. Recently, this fibroblast heterogeneity has become a research topic of interest with respect to the role of fibroblasts in cancer development [3, 4].

In the presence of persistent stimuli, the repair of injured tissue may be accomplished by replacing normal parenchymal tissue with fibrotic tissue. Lung fibrosis often leads to respiratory failure, representing a life-threatening condition. In idiopathic pulmonary fibrosis (IPF), a representative lethal lung fibrotic disease, fibrosis is predominant in the perilobular (subpleural and/or paraseptal) region [5]. To better understand the heterogeneity of fibrosis in IPF, it is first necessary to identify fibroblast subtypes, as well as their localization patterns, and to elucidate their distinct roles. However, molecular markers for human lung fibroblast subtypes have not yet been well established. Therefore, the discovery of a set of markers that may be used to define different human fibroblast subtypes is necessary.

Toward this end, we sought to identify candidate fibroblast markers using microarray analysis of the distinct fibroblasts of the adult mouse lung. Two cell surface markers, CD248 and integrin alpha-8 (ITGA8), were ultimately selected as potentially useful for the identification of human lung fibroblast-like cell subtypes. Based on the expression of these markers, we further evaluated the fibroblast-like cell subtype localization in human samples of normal lungs and the lungs of patients with IPF.

## Methods

### Animal experiments

C57BL/6 female mice (12–16-week-old; 25 g) were purchased from SLC (Shizuoka, Japan). The mice were bred and housed in a pathogen-free mouse facility at a constant temperature and humidity under a 12-h light/12-h dark cycle. All mice were maintained on the sterilized plastic cages containing wood chip bedding and *ad libitium* access to water and food.

### Fluorescence-activated cell sorting (FACS) analysis

The mice were anesthetized by intraperitoneal administration of pentobarbital sodium (77.8 μg/g body mass) (Kyoritsu Seiyaku, Tokyo, Japan) and then sacrificed by CO_2_ asphyxiation. To prepare a single-cell suspension from the mouse lungs for FACS analysis, the lungs were incubated with 200 U/mL of collagenase type 2 (Worthington, Lakewood, NJ, USA) and 100 U/mL DNase I (Worthington) for 30 min at 37 °C in Dulbecco’s phosphate-buffered saline (PBS; Gibco, Carlsbad, CA, USA) [6]. The tissue was cut using gentleMACS Dissociator (Miltenyi Biotechnology, Bergisch Gladbach, Germany). After removing cell aggregates, the obtained suspension was centrifuged at 200×*g* and rinsed twice using FACS buffer (1% HEPES, 2% heat-inactivated fetal calf serum, 120 μg/mL penicillin, and 100 μg/mL streptomycin in Hanks’ buffered salt solution).

In addition to platelet-derived growth factor receptor A (PDGFRA), stem cell antigen-1 (Sca-1) and thymus cell antigen-1 (Thy-1) are used as molecular markers for identifying mouse fibroblasts [6–12]. Single cells were incubated with phycoerythrin (PE)-conjugated anti-PDGFRA antibody, PE-Cy7-conjugated anti-Sca-1 antibody, and PerCp-Cy5.5-conjugated anti-Thy-1.2 antibody; antibodies against lineage-specific cell surface markers allophycocyanin (APC)-conjugated anti-CD31 (vascular endothelial cells), anti-CD45 (hematopoietic cells), anti-CD146 (pericytes and smooth muscle cells), anti-E-cadherin (epithelial cells), anti-LYVE1 (lymphatic endothelial cells), and anti-TER-119 (erythrocytes) (Additional file 1: Table S1); and Sytox Red Dead Cell Stain (1:1000) (Thermo Fisher Scientific, Waltham, MA, USA) for 30 min on ice. After centrifugation (200×*g*) and rinsing of the sample twice in FACS buffer, sorting and analysis were performed using FACSAria (BD Biosciences, San Diego, CA, USA).

To discriminate between ITGA8-positive and ITGA8-negative fibroblasts, single cells were incubated as well with biotin-conjugated anti-ITGA8 antibody (Additional file 1: Table S1) in addition to the antibodies described above. As an isotype control, biotin-conjugated goat IgG antibody (Abcam, Cambridge, UK) was used. After centrifuging (200×*g*) and rinsing twice in FACS buffer, cells were incubated with Alexa 488-conjugated streptavidin (Thermo Fisher Scientific) for 30 min on ice, after which they were centrifuged (200×*g*) and rinsed twice in FACS buffer again, and sorting and analysis were performed using FACSAria.

### Cell culture, colony-forming unit (CFU) assay, cell counting, and immunocytochemistry

Isolated batches of 200 cells in 6-well plates (Grainer bio-one, Kremsmünster, Austria) were grown in Dulbecco’s modified Eagle medium (DMEM; Gibco) supplemented with Glutamax (Gibco), 120 μg/mL penicillin, 100 μg/mL streptomycin, and 20% heat-inactivated fetal calf serum (Gibco) at 37 °C in an atmosphere with 2% O_2_ and 5% CO_2_. The cultured cells were incubated with 1 μg/mL propidium iodide (PI; Sigma-Aldrich, St Louis, MO, USA) for 10 min at 25 °C, fixed in 4% paraformaldehyde for 10 min at 25 °C, and stained with 1 μg/mL Hoechst 33342 (Sigma-Aldrich). For the CFU assay, the colonies consisting of more than 50 viable cells (Hoechst 33342-positive/PI-negative cells) were counted at day 7 of culture. To investigate the proliferative potential, all Hoechst 33342-positive/PI-negative cells in one well of the plates were counted as viable cells in three independent samples in triplicate at days of 1 and 7 of culture. A total of six mice were used for these experiments.

Immunocytochemical staining was performed at day 7 of culture. The adherent cells were fixed in 4% paraformaldehyde for 10 min at 25 °C and permeabilized with 0.5% Triton X-100 in PBS for 5 min at 25 °C. The cells were then incubated in blocking solution (10% goat serum and 0.1% Triton X-100 in PBS) for 30 min at 25 °C, followed by unconjugated anti-αSMA antibody (Additional file 1: Table S1) and 1 μg/mL Hoechst 33342 in blocking solution, and were finally incubated with Alexa-Fluor 488-conjugated anti-mouse IgG_2a_ antibody. Cells were imaged using an Olympus IX71 fluorescence microscope (Olympus). Images were captured using a DP70 camera (Olympus), and subsequently post-processed using Adobe Photoshop CS3.

### Protein quantification of αSMA using FACS

Each fibroblast subpopulation cultured for 7 days was centrifuged; the cells were fixed in 10% buffered formalin for 15 min at 25 °C, and then permeabilized for 5 min using IntraPrep (Beckman Coulter, Brea, CA, USA) according to the manufacturer’s instructions. After centrifuging and rinsing in FACS buffer, the cells were incubated with fluorescein isothiocyanate (FITC)-conjugated anti-αSMA antibody for 30 min at 25 °C. As an isotype control, FITC-conjugated anti-mouse IgG2a (Thermo Fisher Scientific) was used. After centrifuging and rinsing in FACS buffer, the FITC fluorescence intensity of the cells was measured using the FACSAria system. Flow cytometry analysis was performed in triplicate using three independent samples.

### Adipogenic differentiation and osteogenic differentiation

Isolated batches of 800 cells of A- and B-type fibroblasts, and 3000 cells of C-type fibroblasts were grown in 24-well plates (Grainer bio-one) in DMEM supplemented with Glutamax (1:100), 120 μg/mL penicillin, 100 μg/mL streptomycin, and 20% heat-inactivated fetal calf serum at 37 °C in an atmosphere with 2% O_2_ and 5% CO_2_ until they visibly reached confluence. Furthermore, confluent cells were cultured in adipogenic differentiation medium containing 0.5 mM isobutyl-methylxanthine (Sigma-Aldrich), 1 μM dexamethasone (Sigma-Aldrich), and 2.0 μM insulin (Sigma-Aldrich) for 2 days and maintained in medium with 2.0 μM insulin for an additional 2 days. The cells were then maintained in DMEM with 20% fetal calf serum until day 8. The cells were fixed in 4% paraformaldehyde, stained using Oil Red-O (Sigma-Aldrich) to detect cytoplasmic triglycerides, and incubated with unconjugated anti-fatty acid binding protein 4 (FABP4) (Additional file 1: Table S1) and Alexa-Fluor 488-conjugated anti-rabbit IgG antibodies. A total of nine mice were used for these experiments.

Confluent cells were cultured in osteogenic differentiation medium containing 100 nM dexamethasone (Sigma-Aldrich), 0.2 mM ascorbic acid (Sigma-Aldrich), and 10 mM β-glycerophosphate (Sigma-Aldrich) for 21 days. The medium was changed every 3 days, and cells were stained by the von Kossa stain (Sigma-Aldrich) to detect calcium deposition. A total of nine mice were used for this experiment.

### Gene expression profiling

Approximately 7500, 3000, and 7500 A-, B-, and C-type fibroblasts were obtained, respectively, from one mouse. Total RNA was extracted from two independently prepared samples of 50,000 fibroblasts from each of the distinct populations identified by FACS analysis, and from single pulmonary cells (Pu) freshly isolated from the adult mouse lungs using RNeasy Mini Kit (Qiagen, Hilden, Germany). The total RNA was amplified using Ovation PicoSL WTA System V2 (NuGen, San Carlos, CA, USA). Cyanine-3-labeled cDNA was prepared from 2.0 μg DNA using the SureTag Complete DNA Labeling Kit (Agilent, Santa Clara, CA, USA). Cyanine-3-labeled cDNA was hybridized to the Mouse 8 × 60 K (39,430 genes) (Agilent) array. After scanning, the obtained raw data were normalized. The average (log_2_-transformed) gene expression values were calculated, and the obtained values were labeled according to the distinct cell types: Pu, and A-, B-, and C-type fibroblasts (Additional file 1: Table S2–4). The identification strategy used for Sca-1^low^ (C-type fibroblasts) and Sca-1^high^ (A- and B-type fibroblasts) mouse fibroblast-specific genes is described in the footnote to Additional file 1: Table S3 and S4, respectively. A total of 40 mice were used for these experiments.

### Quantitative polymerase chain reaction (qPCR)

The details of the qPCR procedure were described elsewhere [6]. Primer sequences are listed in Additional file 1: Table S5.

### Immunofluorescence staining for adult mouse lungs

After the lung samples obtained from C57BL/6 mice in optimal cutting temperature formulation (Sakura Finetek, Tokyo, Japan) had been frozen at − 80 °C in organic solvent, 6-μm thick slices, sectioned at − 20 °C using a cryostat, were fixed in cold acetone for 10 min and dried for 20 min at 25 °C. The sections were incubated in the blocking solution without detergent (10% goat serum in PBS) for 30 min at 25 °C, after which they were incubated with the appropriate primary antibody (Additional file 1: Table S1) and 1 μg/mL Hoechst 33342 in the blocking solution for 30 min at 25 °C, and were washed in PBS. When anti-ITGA8 antibody (biotin-conjugated) was used, samples were incubated with Alexa-Fluor conjugated streptavidin (Thermo Fisher Scientific). The sections were mounted in Prolong Gold (Thermo Fisher Scientific) and imaged with TSC SP8 confocal microscope (Leica, Wetzlar, Germany). Images were processed with LAS X software (Leica), and then Adobe Photoshop CS3 (Adobe Systems, Inc., San Jose, CA, USA) was used to superimpose three different color images.

### Human tissue samples

From the archives of Hamamatsu University School of Medicine Hospital, we retrieved four human lung samples from autopsy cases without lung disease (normal lungs) and ten human lung biopsy samples by video-assisted thoracic surgery (VATS) from patients with IPF (Additional file 1: Table S6). In addition, we retrieved human skin, large intestine, heart, kidney, and liver samples from autopsy cases. Sequential 4-μm-thick sections from formalin-fixed and paraffin-embedded (FFPE) human lung samples were prepared for hematoxylin and eosin staining, Elastica van Gieson (EVG) staining, conventional immunohistochemistry (IHC), and multiplex immunofluorescence.

### IHC for human samples

Conventional IHC analyses using anti-CD248 and anti-ITGA8 antibodies (Additional file 1: Table S1) were performed after antigen retrieval. The slides were imaged with a BX51 fluorescence microscope (Olympus, Tokyo, Japan). Images were processed with CellSens software (Olympus).

### Multiple immunofluorescence staining for FFPE human lung samples

We used Opal™ 4-color Manual IHC Kit (Perkin Elmer, Waltham, MA, USA), a method for multiplex immunofluorescence staining of FFPE tissues, which uses individual tyramide signal amplification (TSA)-conjugated fluorophores to detect various antigens on one slide. This method enables the use of multiple primary antibodies raised in the same species.

Lung tissue sections were deparaffinized and rehydrated, and the antigen retrieval was performed in a microwave to boil for 20 min using Opal antigen retrieval buffer at pH 6.0. All tissue sections were blocked with antibody diluent for 10 min at 25 °C. The sections were then incubated with pre-diluted anti-lineage-specific markers (CD31, CD45, CD146, D2–40, and E-cadherin) antibodies (Additional file 1: Table S1) for 1 h at 25 °C followed by incubation with horseradish peroxidase (HRP)-conjugated secondary antibodies for 10 min at 25 °C. A 50 × −diluted TSA Plus Working Solution was added to slides and incubated for 10 min at 25 °C. Slides were stripped via microwave, blocked, and then incubated with pre-diluted anti-CD248 or anti-ITGA8 antibody, HRP-conjugated secondary antibodies and TSA Plus Working Solution were used to amplify signals as described above. All primary antibodies were diluted with antibody diluent (Additional file 1: Table S1). Autofluorescence (negative control) slides were also included.

The slides were counterstained with DAPI for 5 min, mounted with Prolong Gold (Thermo Fisher Scientific), and imaged with a TSC SP8 confocal microscope (Leica, Wetzlar, Germany). Images were processed using LAS X software (Leica).

### Quantification of CD248-positive and ITGA8-positive fibroblast-like cells in normal human lungs

In the multiple immunofluorescence for human lungs, we counted 30 lineage-negative cells and calculated the ratio of CD248^high^ITGA8^low^ within lineage-negative cells and CD248^low^ITGA8^high^ fibroblast-like cells within lineage-negative cells in three normal human lungs. In ten IPF samples, we calculated the ratio of CD248-positive and ITGA8-positive fibroblast-like cells as described in Additional file 1: Figure S1.

### Statistical analysis

The obtained results are presented as mean values ± standard deviations of the results obtained in at least three independent experiments. Statistical analyses were performed using an unpaired Student’s *t*-test (two-tailed) for comparisons between two groups, and with one-way analysis of variance and Bonferroni correction for comparisons between more than two groups. *P* < 0.05 was considered to represent statistical significance.

## Results

### Isolation of immunophenotypically distinct fibroblasts from the mouse lungs by FACS

**Fig. 1:**
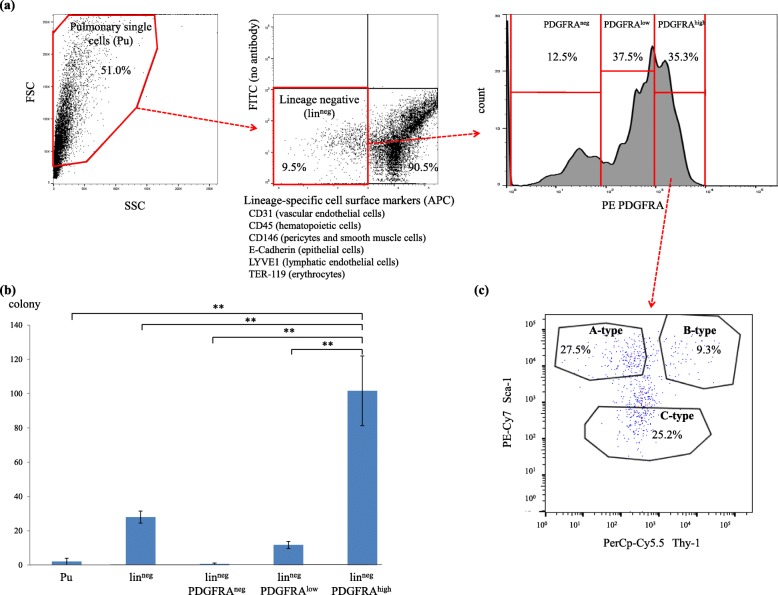
**a** *Left*: Flow cytometry side scatter (SSC: x-axis) vs. forward scatter (FSC: y-axis) plot, showing single cells isolated from the mouse lung. *Middle*: expression levels of lineage-specific cell surface markers on these cells (x-axis); Lin^neg^ cells were sorted from the APC-negative fraction (red rectangle). *Right*: Histogram showing platelet-derived growth factor receptor A (PDGFRA) expression levels on lin^neg^ cells (x-axis); representative results are presented in all panels. **b** Two hundred cells of each indicated population were cultured. The numbers of colonies developing from pulmonary single cells (Pu), lin^neg^, lin^neg^PDGFRA^neg^, lin^neg^PDGFRA^low^, and lin^neg^PDGFRA^high^ cells at day 7 after sorting are presented. All experiments were performed in triplicate, and means ± standard deviations of the results obtained in three independent experiments are presented; **P < 0.01. **c** Representative flow cytometry plot showing the expression of Thy-1 and Sca-1 on lin^neg^PDGFRA^high^ cells. A-, B-, and C-type fibroblasts indicate lin^neg^PDGFRA^high^Sca-1^high^Thy-1^low^, lin^neg^PDGFRA^high^Sca-1^high^Thy-1^high^, and lin^neg^PDGFRA^high^Sca-1^low^ cells, respectively

Through FACS analysis, using antibodies against six lineage-specific cell surface markers (lin) (Additional file 1: Table S1), we isolated lin^neg^ cells from the mouse lungs; this cell population comprised a small number of lineage-committed cells, including vascular endothelial cells, hematopoietic cells, pericytes, smooth muscle cells, epithelial cells, lymphatic endothelial cells, and erythrocytes (Fig. 1a, left and middle panels). Furthermore, cells were distinguished based on the expression levels of PDGFRA, PDGFRA^neg^, PDGFRA^low^, and PDGFRA^high^ cells (Fig. 1a, right panel). The number of CFUs in the lin^neg^PDGFRA^high^ group was considerably higher than that in the other cultured cell types (Fig. 1b).

In addition to PDGFRA, Sca-1 and Thy-1 are also used as molecular markers for identifying mouse fibroblasts [6–12]. As shown in Fig. 1c, FACS analysis separated fibroblasts into Sca-1^high^Thy-1^low^ (A-type fibroblasts) and Sca-1^high^Thy-1^high^ (B-type fibroblasts) from lin^neg^PDGFRA^high^ cells. We additionally identified another fibroblast subpopulation, Sca-1^low^ (C-type fibroblasts) that has not been reported to date.

### In vitro characterization of immunophenotypically distinct fibroblasts from the mouse lungs

**Fig. 2:**
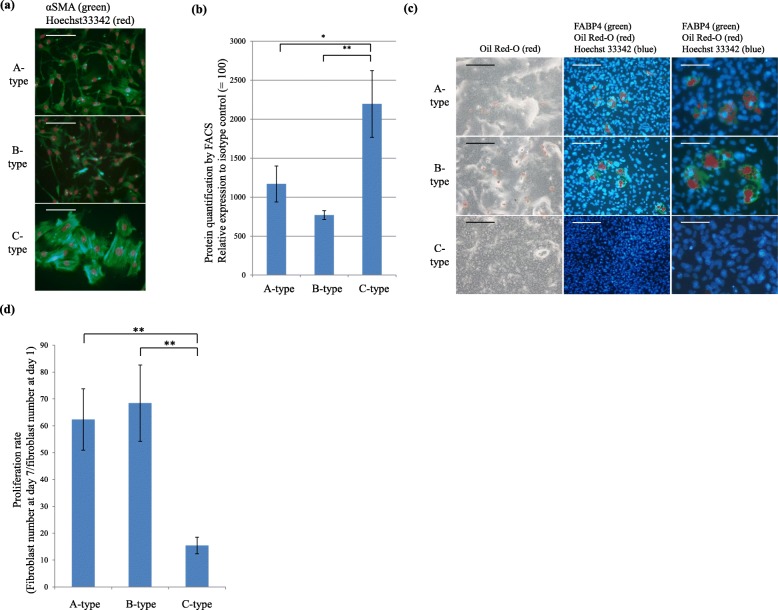
**a** Two hundred fibroblasts of the indicated type were cultured for 7 days and stained with Hoechst 33342 (red, nucleus) and anti-αSMA antibody (green); merged images are shown and representative results are presented. Scale bars, 50 μm. **b** Protein levels of intracellular αSMA in A-, B-, and C-type fibroblasts cultured for 7 days determined by FACS using FITC-conjugated anti-αSMA antibody, measured relative to the level using FITC-conjugated mouse IgG2a antibody as a control. The experiments were performed in triplicate, and the mean ± SD of the results obtained in three independent experiments are presented. *P < 0.05, ** *P* < 0.01. **c** All fibroblast types were cultured until confluence, followed by incubation in the adipogenic differentiation medium for 14 days and stained with Hoechst 33342 (blue, *middle and right*), anti-fatty acid binding protein 4 (FABP4) antibody (green, *middle and right*), and Oil Red-O (red, *all panels*). Merged images are shown (*middle and right*) and representative results are presented. Scale bars, *left, middle* 50 μm; *right,* 25 μm. **d** Proliferation rates of different fibroblast types (fibroblast number at day 7/fibroblast number at day 1). Data represent mean values ± standard deviations of the results obtained from three independent experiments performed in triplicate; **P < 0.01

Staining of the fibroblasts with anti-αSMA antibody after 7 days of culture demonstrated that A- and B-type fibroblasts consisted of spindle fibroblast colonies, whereas C-type fibroblasts consisted of polygonal fibroblast colonies with many smooth muscle fibers (Fig. 2a). Protein quantification using FACS revealed significantly higher expression levels of αSMA in C-type fibroblasts than in the A- and B-type fibroblasts (Fig. 2b).

Next, we investigated the ability of the isolated fibroblasts to differentiate into osteocytes and adipocytes. Although the cells were cultured in osteogenic differentiation medium, no osteoblast differentiation was observed in any of the cultures, indicating that they were not mesenchymal stem cells (Additional file 1: Figure S2). However, in the adipogenic differentiation medium, some A- and B-type fibroblasts showed lipid droplets and expressed FABP4, which is a specific marker for adipocytes. In contrast, no adipocyte differentiation of C-type fibroblasts was observed (Fig. 2c).

To investigate the proliferative capacity of the different fibroblast subpopulations, we determined the number of fibroblasts after day 1 and day 7 of incubation and calculated the respective proliferation rates. After 7 days in culture, the proliferation rates of A- and B-type fibroblasts were similar and significantly higher than those of C-type fibroblasts (Fig. 2d). In addition, cell cycle analysis using 5-ethynyl-2′-deoxyuridine demonstrated that the rapid cell cycle progression of A- and B-type fibroblasts resulted in their faster proliferation (Additional file 1: Figure S3).

These results indicated that A- and B-type fibroblasts proliferated rapidly, exhibiting partial differentiation into adipocytes, whereas C-type fibroblasts proliferated slowly, and exhibited more myofibroblastic differentiation but did not show adipocyte differentiation in vitro.

### Gene expression profiling of different fibroblast subtypes from the mouse lungs

**Fig. 3:**
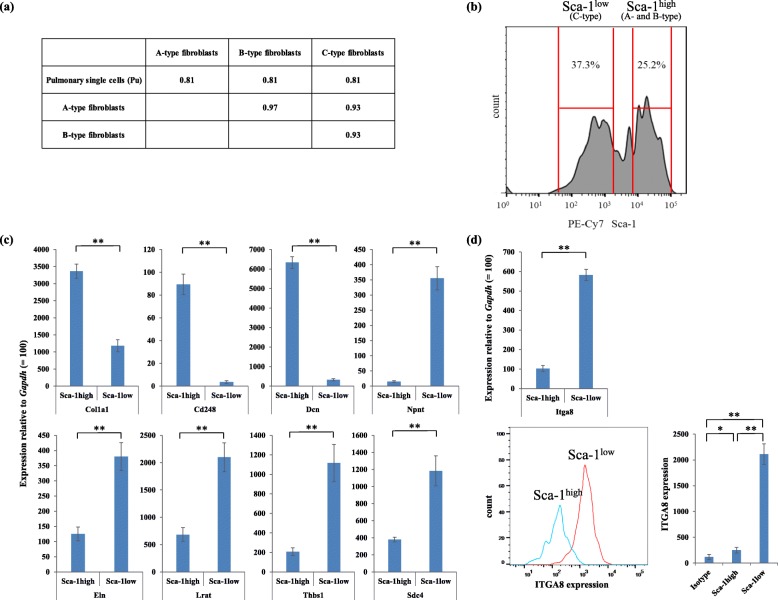
**a** Pearson correlation coefficients (r^2^) of the microarray data between two samples were calculated using the average (log_2_-transformed) gene expression values. **b** Representative flow cytometry plot showing the expression of Sca-1 on lin^neg^PDGFRA^high^ cells (see Fig. 1a). Sca-1^high^ fibroblasts and Sca-1^low^ fibroblasts are indicated. Quantitative PCR for (**c**) collagen 1a1 (*Col1a1*), *Cd248*, decorin (*Dcn*), nephronectin (*Npnt*), elastin (*Eln*), lecithin-retinol acyltransferase (*Lrat*), thrombospondin (*Thbs1*), and syndecan 4 (*Sdc4*) were performed using prepared cDNA samples of the freshly isolated Sca-1^high^ and Sca-1^low^ fibroblasts, measured relative to the level of glyceraldehyde-3-phosphate dehydrogenase (*Gapdh*) for each sample, which was adjusted to 100. Three independent experiments were performed in triplicate. Mean values ± standard deviations are presented; **P < 0.01. **d** Upper: Quantitative PCR for integrin alpha-8 (*Itga8*). Lower left: Representative flow cytometry plot showing the expression of ITGA8 on Sca-1^high^ fibroblasts (blue line) and Sca-1^low^ fibroblasts (red line). Lower right: Quantitative analysis of ITGA8 expression by FACS. Isotype indicates flow cytometric analysis using biotin-conjugated goat antibody as a control. Three independent experiments were performed in triplicate. Mean values ± standard deviations are presented; **P < 0.01

Because the levels of gene expression drastically changed during the culture (Additional file 1: Figure S4), we used mouse lung fibroblasts freshly isolated by FACS to identify any genes specific to each fibroblast subtype. Following amplification of the mRNA of 50,000 cells of each fibroblast subpopulation (A-, B-, and C-type), microarray analysis was performed to compare the gene expression profiles (Additional file 1: Table S2). The gene expression patterns of A- and B-type fibroblasts were similar (Fig. 3a), corresponding to the lack of any significant in vitro biological differences (Fig. 2). Therefore, we did not need to further discriminate between A- and B-type fibroblasts, and they were subsequently classified together into a single fibroblast subtype termed Sca-1^high^ mouse fibroblasts. Thus, the gene expression profiles of Sca-1^high^ mouse fibroblasts and Sca-1^low^ mouse fibroblasts (C-type fibroblasts) were further compared.

According to our identification strategy for Sca-1^high^ and Sca-1^low^ mouse fibroblast-specific genes, 23 genes were upregulated in Sca-1^low^ mouse fibroblasts, whereas 36 genes were upregulated in Sca-1^high^ mouse fibroblasts (Additional file 1: Table S3 and S4). Furthermore, we performed qPCR assay of nine genes that are listed in Additional file 1: Table S3 and S4 to validate the obtained microarray data (Fig. 3c).

### ITGA8 is a cell surface marker for Sca-1^low^ mouse lung fibroblasts

Because the amount of freshly isolated fibroblasts from the lungs was too small to be used for western blotting, the obtained microarray data were validated by FACS-based protein quantification assay in addition to qPCR assay.

It was necessary to identify specific cell surface markers of Sca-1^low^ mouse fibroblasts. The genes listed in Additional file 1: Table S3 and qPCR results (Fig. 3d) suggested that integrin alpha8 (*Itga8*) gene was differentially expressed between Sca-1^high^ and Sca-1^low^ mouse fibroblasts. Furthermore, FACS-based protein quantification assay confirmed that the expression levels of ITGA8 were significantly higher in Sca-1^low^ mouse fibroblasts than in Sca-1^high^ mouse fibroblasts (Fig. 3d).

### Localization of Sca-1^high^ and Sca-1^low^ fibroblasts in mouse lungs

**Fig. 4:**
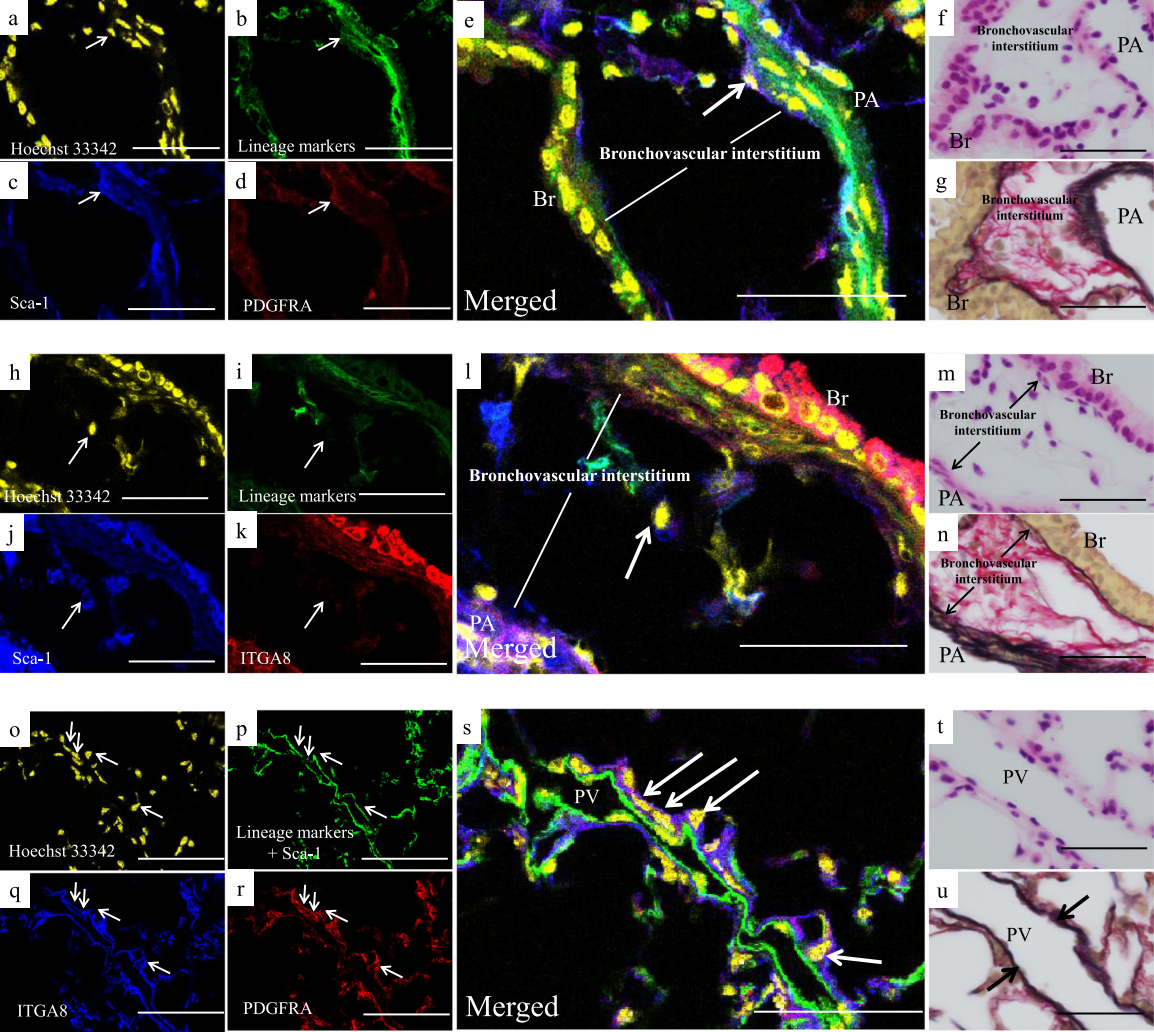
Localization of Sca-1^high^ fibroblasts and Sca-1^low^ fibroblasts in adult mouse lungs. Hoechst 33342 (**a**), lineage-specific cell surface markers (CD31, CD45, CD146, E-cadherin, LYVE1, and TER-119) (**b**), Sca-1 (**c**), and PDGFRA (**d**) staining, merged images (**e**). Mouse lungs were also stained with hematoxylin and eosin (H&E) (**f**) and Elastica van Gieson stain (EVG) (**g**); collagen fibers are indicated as red bundles, and elastic fibers are indicated as black bundles. Arrows in (**a**) to (**e**) indicate Sca-1^high^lin^neg^PDGFRA^high^ fibroblasts localized in the adventitia of pulmonary artery. Hoechst 33342 (**h**), lineage-specific cell surface markers (**i**), Sca-1 (**j**), and ITGA8 (**k**) staining, merged images (**l**). Mouse lungs were also stained with H&E (**m**) and EVG (**n**). Arrows in (**h**) to (**l**) indicate Sca-1^high^lin^neg^ITGA8^low^ fibroblasts localized in the bronchovascular interstitium. Hoechst 33342 (**o**), lineage-specific cell surface markers and Sca-1 (**p**), ITGA8 (**q**), and PDGFRA (**r**) staining, merged images (**s**). Mouse lungs were also stained with H&E (**t**) and EVG (**u**). Arrows in (**o**) to (**s**) indicate Sca-1^low^lin^neg^ITGA8^high^ fibroblasts localized in the adventitia of pulmonary vein. Arrows in (**u**) indicate elastic fibers in the adventitia of pulmonary vein. Br, bronchial epithelium. PA, pulmonary artery. PV, pulmonary vein. Representative results are presented in all panels; scale bars, 50 μm

To visualize the localization of Sca-1^low^ fibroblasts and Sca-1^high^ fibroblasts in mouse lungs, we used antibodies against the lineage-specific cell surface markers (lin), PDGFRA, Sca-1, and ITGA8. Immunofluorescence staining demonstrated that the Sca-1^high^ITGA8^low^ mouse fibroblasts were localized in the adventitia of pulmonary artery and bronchovascular interstitium, which were the most noticeable collagen fiber (red fiber in EVG stain)-rich connective tissue in mouse lungs (Fig. 4a–n), whereas Sca-1^low^ITGA8^high^ mouse fibroblasts were localized in the adventitia of pulmonary vein, which were the most noticeable elastic fiber (black fiber in EVG stain)-rich connective tissue in mouse lungs (Fig. 4). These results indicated that the two immunophenotypically distinct mouse fibroblast subtypes were differently localized in lungs.

As there is no human homolog of mouse Sca-1, it was necessary to identify specific molecular markers of Sca-1^high^ mouse fibroblasts. The genes listed in Additional file 1: Table S4 suggested that Cd248 may be differentially expressed between Sca-1^high^ and Sca-1^low^ mouse fibroblasts. The qPCR assay confirmed that the expression levels of CD248 were much higher in Sca-1^high^ mouse fibroblasts than in Sca-1^low^ mouse fibroblasts (Fig. 3c). However, in the present study, we were unable to obtain high-quality results for FACS and immunofluorescence analysis of the mouse lungs using the commercial antibodies against mouse CD248.

The characteristics of the immunophenotypically distinct fibroblast subtypes of mouse lungs are summarized in Table 1.

**Table 1:** Characteristics of immunophenotypically different fibroblast subtypes in adult mouse lungs

Type of fibroblast	Culture cell morphology	Proliferation	Adipocyte differentiation	*Cd248* expression	ITGA8 expression	*Col1a1* expression	*Eln* expression	Localization in mouse lung
Sca-1^high^	Spindle	Rapid	+	High	Low	High	Low	Collagen fiber-rich connective tissue
Sca-1^low^	Flat	Slow	–	Low	High	Low	High	Elastic fiber-rich connective tissue

+: Recognized, −: Not-recognized

### Localization of different immunophenotypical fibroblast-like cells in normal human lungs

**Fig. 5:**
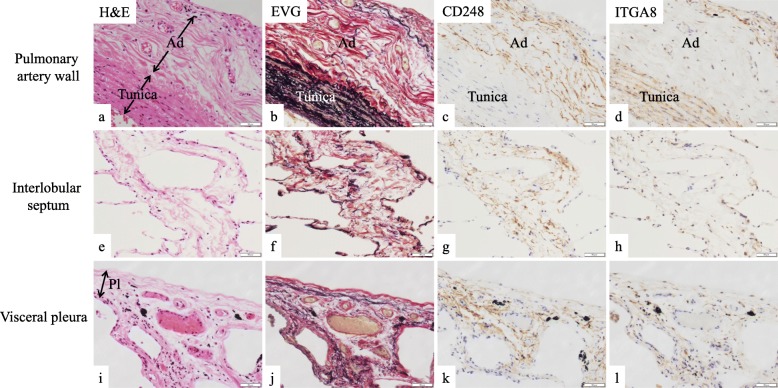
Serial sections of normal human lungs were stained with (**a, e, i**) hematoxylin and eosin (H&E) and (**b**, **f**, **j**) Elastica van Gieson stain (EVG); collagen fibers are indicated as red bundles, and elastic fibers are indicated as black bundles. Immunohistochemistry (IHC) of normal human lungs using (**c**, **g**, **k**) anti-CD248 antibody and (**d**, **h**, **l**) anti-ITGA8 antibody. (**a**–**d**) Pulmonary artery wall (tunica indicates the smooth muscle layer of the pulmonary artery, Ad indicates the adventitia of pulmonary artery). (**e**–**h**) Interlobular septum. (**i**–**l**) Visceral pleura. Pl, visceral pleura. Representative results are presented in all panels; scale bars, 50 μm

**Fig. 6:**
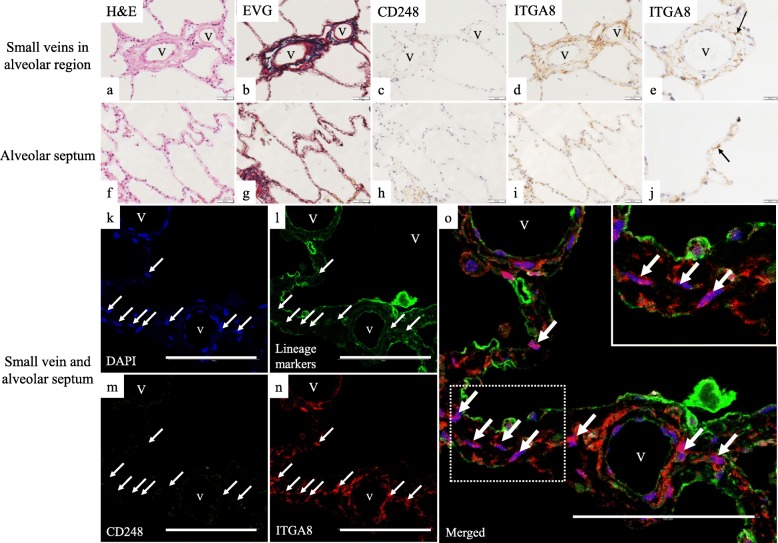
Serial sections of normal human lungs were stained with (**a** and **f**) hematoxylin and eosin (H&E) and (**b** and **g**) Elastica van Gieson stain (EVG). IHC of normal human lungs using (**c** and **h**) anti-CD248 antibody and (**d**, **e**, **i**, **j)** anti-ITGA8 antibody; **a**–**e**, small veins in alveolar region and **f**–**j**, alveolar septum. Multiplex immunofluorescent images of the small veins and the alveolar septum: (**k**) blue, DAPI; (**l**) green, lineage-specific markers; (**m**) yellow, CD248; (**n**) red, ITGA8; and (**o**) merged. White arrows in (**k**–**o**) indicate the nuclei of lin^neg^CD248^low^ITGA8^high^ fibroblast-like cells located in the adventitia of the small vein and in the alveolar septum. Inset in panel (**o**) is an enlarged figure of the dotted rectangle of the alveolar septum; the white arrows indicate the nuclei of lin^neg^CD248^low^ITGA8^high^ fibroblast-like cell shown in the inset. V, small vein. Representative results are presented in all panels; scale bars, 50 μm (**a**–**d**, **f**–**i**), 20 μm (**e** and **j**), 100 μm (**k**–**o**)

**Fig. 7:**
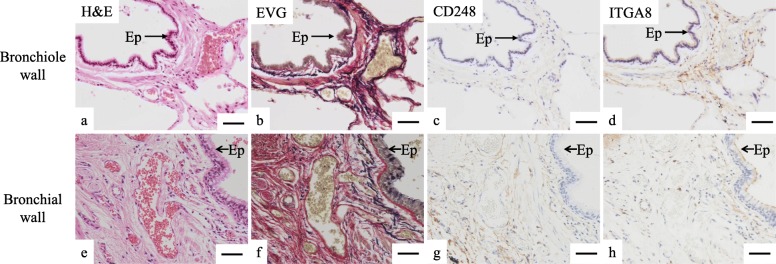
Serial sections of normal human lungs were stained with (**a** and **e**) hematoxylin and eosin (H&E) and (**b** and **f**) Elastica van Gieson stain (EVG). IHC of normal human lungs using (**c** and **g**) anti-CD248 and (**d** and **h**) anti-ITGA8 antibody: **a**–**d**, bronchiole wall; **e**–**h**, bronchial wall. Ep, Epithelial cells. Representative results are presented in all panels; scale bars, 50 μm

To reveal the localization of distinct fibroblast subtypes in the normal human lungs, we performed conventional IHC analysis using anti-CD248 antibody or anti-ITGA8 antibody. Further, we conducted multiplex immunofluorescence (Opal®) using antibodies against lineage-specific markers (CD31 for endothelial cells, CD45 for hematopoietic cells, CD146 for pericytes and smooth muscle cells, D2–40 for lymphatic endothelial cells, and E-cadherin for epithelial cells) in addition to anti-CD248 and anti-ITGA8 antibodies in order to detect lineage-specific markers-negative cells (mostly fibroblasts). As expected, two immunophenotypically different fibroblast-like cells—lineage-specific marker-negative/CD248-high/ ITGA8-low (CD248^high^ITGA8^low^) fibroblast-like cells and lineage-specific marker-negative/CD248-low/ ITGA8-high (CD248^low^ITGA8^high^) fibroblast-like cells—were shown to be major subtypes in normal human lungs (Fig. 5a–l, Fig. 6a–j, Fig. 7a–h).

**Fig. 8:**
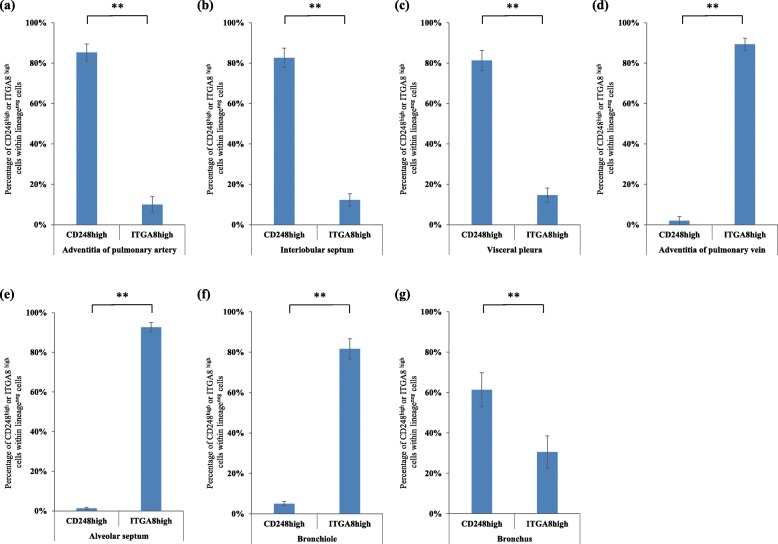
Quantification of CD248^high^ITGA8^low^ fibroblast-like cells and CD248^low^ITGA8^high^ fibroblast-like cells in the seven regions of normal human lungs (*n* = 4) in the multiplex immunofluorescence staining. The ratio of CD248^high^ITGA8^low^ fibroblast-like cells within lineage-specific markers-negative cells (30 cells) and CD248^low^ITGA8^high^ fibroblast-like cells within lineage- specific markers-negative cells (30 cells) were calculated. (**a**) Adventitia of pulmonary artery, (**b**) Interlobular septum, (**c**) Visceral pleura, (**d**) Adventitia of pulmonary vein, (**e**) Alveolar septum, (**f**) Bronchiole wall, (**g**) Bronchial wall. CD248high and ITGA8high (**a**–**g**) indicate CD248^high^ITGA8^low^ fibroblast-like cells and CD248^low^ITGA8^high^ fibroblast-like cells, respectively. All experiments were performed in triplicate, and means ± standard deviations of the results obtained in three independent experiments are presented; **P < 0.01

The multiplex immunofluorescence followed by the quantification of the number of two subtypes demonstrated that CD248^high^ITGA8^low^ human fibroblast-like cells were predominantly distributed in the adventitia of the pulmonary artery (Fig. 8a and Additional file 1: Figure S5), interlobular septum (Fig. 8b), and visceral pleura (Fig. 8c and Additional file 1: Figure S6), which are collagen fiber (red fibers in EVG stain)-rich connective tissue (Fig. 5a–l). By contrast, CD248^low^ITGA8^high^ human fibroblast-like cells were localized in the adventitia of the pulmonary vein (Fig. 6k–o and Fig. 8d) and in the alveolar septum (Fig. 6 k–o and Fig. 8e), which are elastic fiber (black fibers in EVG stain)-rich connective tissues (Fig. 6a–j).

The distribution of fibroblast-like cell subtypes in the airway wall varied among the different subtypes. CD248^low^ITGA8^high^ human fibroblast-like cells were the major subtype identified in the bronchiole wall, which are elastic fiber-rich connective tissues (Fig. 7a–d and Fig. 8f). By contrast, CD248^high^ITGA8^low^ human fibroblast-like cells were the major subtype identified in the bronchial wall, which are collagen fiber-rich connective tissue (Fig. 7e–h and Fig. 8g).

### Localization of CD248^high^ITGA8^low^ fibroblast-like cells and CD248^low^ITGA8^high^ fibroblast-like cells in other major human organs

In other major human organs, we immunohistochemically examined the expression of CD248 and ITGA8 in fibroblast-like cells (Additional file 1: Figure S7). In the human dermis, which contains abundant collagen and elastic fibers, conventional IHC demonstrated that dermal fibroblast-like cells expressed CD248 but not ITGA8. Only CD248^high^ fibroblast-like cells were found in intestine and heart like dermis, whereas neither CD248^high^ nor ITGA8^high^ fibroblast-like cells were found in kidney or liver. During our examination, we did not find CD248^low^ITGA8^high^ human fibroblast-like cells in other major organs. Thus, the lung is the only organ where CD248^high^ITGA8^low^ and CD248^low^ITGA8^high^ human fibroblast-like cells coexisted and were differently localized.

### Localization of CD248^high^ITGA8^low^ fibroblast-like cells and CD248^low^ITGA8^high^ fibroblast-like cells in IPF lungs

**Fig. 9:**
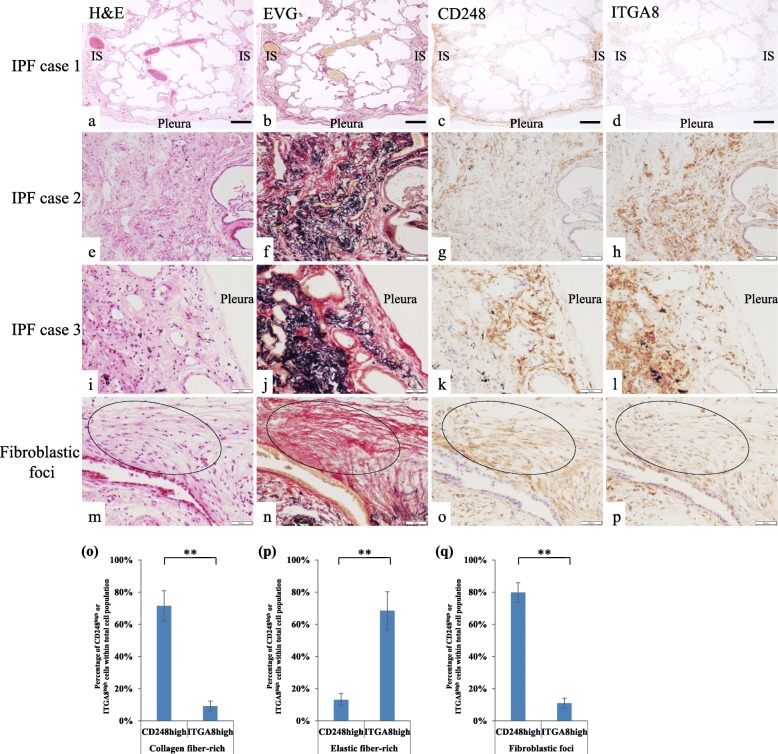
Serial tissue sections of IPF lungs (cases 1–3) were stained with (**a**, **e**, **i**, **m**) hematoxylin and eosin (H&E) and (**b**, **f**, **j**, **n**) Elastica van Gieson stain (EVG). IHC of IPF lungs using (**c**, **g**, **k**, **o**) anti-CD248 antibody and (**d**, **h**, **l**, **p**) anti-ITGA8 antibody. In panels **a**–**d**, a lobule is located between two interlobular septa (IS). Note that fibrosis was predominant in the IS and subpleural area. (**m**–**p**) IHC of the fibroblastic foci of the IPF lung. Dotted eclipses indicate fibroblastic foci. Representative results are presented in all panels; scale bars, 250 μm (**a**–**d**), 50 μm (**e**–**p**). Quantitative analysis of CD248^high^ fibroblast-like cells and ITGA8^high^ fibroblast-like cells in collagen fiber-rich connective tissue **(o)**, elastic fiber-rich connective tissue (**p**), and fibroblastic foci (**q**) in IPF lungs (*n* = 10), respectively (See Additional file 1: Figure S1). Because the number of the nuclei of other cells types such as pericytes, epithelial cells, and leukocytes were included in the total cell number, the percentage of CD248^high^ fibroblast-like cells + ITGA8^high^ fibroblast-like cells did not reach 100% in Fig. 9o–q. All experiments were performed in triplicate, and means ± standard deviations of the results obtained in three independent experiments are presented; **P < 0.01

We examined the expression of CD248 and ITGA8 on fibroblast-like cells in IPF lungs by conventional IHC. Figure 9a-d shows a lung lobule in which fibrosis was predominant in the subpleural and paraseptal region, with relatively less fibrosis inside the lobule. Many collagen fibers and CD248^high^ITGA8^low^ human fibroblast-like cells were found in the same fibrotic regions with a few CD248^low^ITGA8^high^ human fibroblast-like cells detected (Fig. 9a–d), and the expression levels of CD248 in the IPF lungs were significantly increased compared with those in the normal lungs (Additional file 1: Figure S8). In the fibrotic regions of all cases, CD248^high^ITGA8^low^ human fibroblast-like cells were also localized in the collagen fiber-rich connective tissue (Fig. 9a–l and o), whereas CD248^low^ITGA8^high^ human fibroblast-like cells were localized in the elastic fiber-rich fibrotic connective tissue (Fig. 9e–l and p). CD248^high^ITGA8^low^ human fibroblast-like cells were found in the collagen fiber-rich connective tissue of fibroblastic foci, a hallmark of IPF (Fig. 9m–p and q).

## Discussion

The cell surface marker Sca-1, which has been used to identify immunophenotypically distinct mouse lung fibroblasts, is expressed in stem/progenitor cells of various mouse tissues, such as skeletal system, mammary glands, prostate, dermis, skeletal muscle, heart, and liver, in addition to fibroblasts [13]. Sca-1^high^ and Sca-1^low^ fibroblast subtypes were found in skin, bone marrow, and thymus [14], but the molecular mechanism of different Sca-1 expression levels between Sca-1^high^ and Sca-1^low^ fibroblast subtypes and the biological function of Sca-1 on fibroblasts has not been elucidated.

However, by using multiple antibodies, including anti-Sca-1 antibody, we identified two distinct mouse lung fibroblast subtypes, including a newly identified subtype (Sca-1^low^), which differed from the other subtype (Sca-1^high^) in terms of the in vitro characteristics such as the cultured cell morphology (Fig. 2a), proliferation (Fig. 2b), differentiation (Fig. 2c), and gene expression profiles (Fig. 3). Interestingly, we revealed that the Sca-1^high^ mouse fibroblasts and their corresponding CD248^high^ITGA8^low^ human fibroblast-like cells were localized in the collagen fiber-rich connective tissue, whereas Sca-1^low^ mouse fibroblasts and their corresponding CD248^low^ITGA8^high^ human fibroblast-like cells were localized in the elastic fiber-rich connective tissue in normal mouse/human lungs (Fig. 4–8). These IHC results were consistent with the qPCR results of the expression levels of *Col1a1* and *Eln* in Sca-1^high^ and Sca-1^low^ mouse fibroblasts (Fig. 3c).

The expression of CD248 and Sca-1 was negatively regulated, and the expression of ITGA8 was positively regulated by transforming growth factor (TGF)-β, which plays a critical role in the progression of fibrosis of IPF [15–18], suggesting that expression of CD248 and ITGA8 may be potentially changed in the pro-fibrotic state. However, like normal human lungs, the CD248^high^ITGA8^low^ human fibroblast-like cells were localized in collagen fiber-rich connective tissue, and CD248^low^ITGA8^high^ human fibroblast-like cells were localized in elastic fiber-rich connective tissue (Fig. 9), demonstrating that this classification system of human lung fibroblasts could be used even in IPF lungs.

CD248 is a receptor for type I collagen, which consists of collagen fibers in the connective tissue [19–22]. Therefore, it seems reasonable that CD248^high^ITGA8^low^ fibroblast-like cells were always localized in the collagen fiber-rich connective tissues (Figs. 5, 7, 8, and 9). Bartis et al. [23] reported that CD248 was expressed in the fibroblast-like cells in the fibrotic region of IPF lungs like our results and that the expression levels of CD248 were positively correlated with IPF severity. As shown in the previous study [23], severe IPF lung samples were obtained from lung transplant patients with severe stage IPF. However, we obtained lung samples by VATS, which is generally conducted in mild to moderate stage IPF patients but not in severe stage IPF patients (Additional file 1: Table S6). Therefore, we could not investigate the correlation between severity of IPF and the expression of CD248 and ITGA8 in this study.

CD248 was expressed in the fibroblastic foci (Fig. 9), which histologically characterize IPF and are located in the interface between the fibrotic and the normal parenchyma of the pulmonary lobule [24]. The amount of fibroblastic foci is a significant prognostic factor of IPF [25], and fibroblastic foci itself may be a potential therapeutic target of IPF. Because CD248^high^ITGA8^low^ human fibroblast-like cells may play an important role in the formation of fibroblastic foci as well as fibrosis in the perilobular (subpleural and/or paraseptal) region (Fig. 9a-d), controlling those fibroblasts may lead to the suppression of fibrosis of IPF. Bartis et al. and Benedetto et al. demonstrated that the reduction of CD248 expression on the fibroblast cell line via siRNA resulted in the reduction of in vitro cell proliferation [23, 26], suggesting that CD248^high^ITGA8^low^ human fibroblast-like cells are possible candidates as therapeutic targets for IPF lungs. As an anti-fibrotic therapy, CD248 blocking peptide as well as siRNA may be effective tools to suppress the function or expression of CD248 on CD248^high^ITGA8^low^ human fibroblast-like cells without affecting CD248^low^ITGA8^high^ human fibroblast-like cells.

ITGA8 was expressed in fibroblast-like cells of the alveolar region of normal human lungs, consistent with previous reports [27, 28]. *Itga8*^−/−^ mice have been reported to show pronounced aberrations in development of the normal lung structure, as evidenced by the presence of wavy and short elastic fibers [29]. In addition, Hung et al. recently demonstrated that fibrotic stimuli by administration of bleomycin induced lung fibrosis in mice in which Itga8 was genetically deleted on platelet-derived growth factor beta-positive stromal cells as well as in the control mice, suggesting that ITGA8 was not involved in the collagen production of the mouse lung fibrosis model [30]. These results seem to be consistent with the results that Sca-1^low^ mouse fibroblasts and their corresponding CD248^low^ITGA8^high^ human fibroblast-like cells were localized in the elastic fiber-rich connective tissue but not in collagen fiber-rich connective tissue in normal and IPF lungs (Figs. 6, 7, 8, and 9).

In this study, it was revealed that the distribution of two human lung fibroblasts subtypes was consistent with that of collagen and elastic fibers. Further functional evidence is needed to demonstrate that two fibroblast subsets play an important role in the extracellular matrix deposition and remodeling in fibrotic lungs.

## Conclusions

We identified CD248 and ITGA8 as cell surface markers for human lung fibroblast subtypes. Given their localization, two major subtypes, CD248^high^ITGA8^low^ human fibroblast-like cells and CD248^low^ITGA8^high^ human fibroblast-like cells, appear to be associated with collagen fibers and elastic fibers, respectively. This classification system of human lung fibroblasts may be used to conduct further detailed investigations of the functions of different fibroblasts, which will help to gain new insights into lung development and the pathological processes underlying IPF and other fibrotic lung diseases such as pleuroparenchymal fibroelastosis, non-specific interstitial pneumonia, and organizing pneumonia.

## Supplementary information

**Additional file 1 Figure S1.** Quantification of CD248-positive fibroblast-like cells and ITGA8-positive fibroblast-like cells in collagen fiber-rich connective tissue and elastic fiber-rich connective tissue in IPF lungs. **Figure S2.** Osteoblast differentiation in mouse fibroblast subtypes. **Figure S3.** Cell cycle of mouse fibroblast subtypes were analyzed using 5-ethynyl-2′-deoxyuridine (EdU). **Figure S4.** Expression levels of *Sca-1* and *Itga8* during mouse fibroblast culture. **Figure S5.** Multiple immunofluorescence (IF) images of pulmonary artery wall (media and adventitia) of normal human lung. **Figure S6.** Multiple IF images of visceral pleura of normal human lung. **Figure S7.** Localization of CD248^high^ITGA8^low^ fibroblast-like cells and CD248^low^ITGA8^high^ fibroblast-like cells in other major human organs. **Figure S8.** Graphical description of the digital image analysis using the Color Deconvolution ImageJ plugin. **Table S1.** Details of the antibodies used in this study. **Table S2.** Microarray analysis results of pulmonary single cells and three immunophenotypically distinct mouse fibroblast types. **Table S3.** Specific markers associated with Sca-1^low^ mouse fibroblast (C-type fibroblast)-specific genes. **Table S4.** Specific markers associated with Sca-1^high^ mouse fibroblast (A- and B-type fibroblast)-specific genes. **Table S5.** Primers used for quantitative PCR used in this study. **Table S6.** The demographic and clinical data of 10 patients with histologically confirmed IPF.

## Data Availability

All relevant data and materials are published in the manuscript and supplementary materials.

## Abbreviations

CFU: Colony-forming unit
*Col1a1*: Collagen 1a1
*Dcn*: Decorin
*Eln*: Elastin
EVG: Elastica van Gieson
FABP4: Fatty acid binding protein 4
FACS: Fluorescence-activated cell sorting
H&E: Hematoxylin and eosin
IPF: Idiopathic pulmonary fibrosis
ITGA8: Integrin alpha-8
lin: Lineage-specific markers
*Lrat*: Lecithin-retinol acyltransferase
*Npnt*: Nephronectin
PDGFRA: Platelet-derived growth factor receptor type-A
*Sdc4*: Syndecan 4
*Thbs1*: Thrombospondin
