# Supplementary material for: CD248 and integrin alpha-8 are candidate markers for differentiating lung fibroblast subtypes
Source: BMC Pulm Med. 2020 Jan 21;20:21. doi: 10.1186/s12890-020-1054-9 (PMC6975017; doi:10.1186/s12890-020-1054-9)
Supplement: Supplementary file 1 — Additional file 1 Figure S1. Quantification of CD248-positive fibroblast-like cells and ITGA8-positive fibroblast-like cells in collagen fiber-rich connective tissue and elastic fiber-rich connective tissue in IPF lungs. Figure S2. Osteoblast differentiation in mouse fibroblast subtypes. Figure S3. Cell cycle of mouse fibroblast subtypes were analyzed using 5-ethynyl-2′-deoxyuridine (EdU). Figure S4. Expression levels of Sca-1 and Itga8 during mouse fibroblast culture. Figure S5. Multiple immunofluorescence (IF) images of pulmonary artery wall (media and adventitia) of normal human lung. Figure S6. Multiple IF images of visceral pleura of normal human lung. Figure S7. Localization of CD248highITGA8low fibroblast-like cells and CD248lowITGA8high fibroblast-like cells in other major human organs. Figure S8. Graphical description of the digital image analysis using the Color Deconvolution ImageJ plugin. Table S1. Details of the antibodies used in this study. Table S2. Microarray analysis results of pulmonary single cells and three immunophenotypically distinct mouse fibroblast types. Table S3. Specific markers associated with Sca-1low mouse fibroblast (C-type fibroblast)-specific genes. Table S4. Specific markers associated with Sca-1high mouse fibroblast (A- and B-type fibroblast)-specific genes. Table S5. Primers used for quantitative PCR used in this study. Table S6. The demographic and clinical data of 10 patients with histologically confirmed IPF. [file 12890_2020_1054_MOESM1_ESM.docx]

**Additional file 1**

**CD248 and integrin alpha-8 are candidate markers for differentiating lung fibroblast subtypes**

Sayomi Matsushima^1,2^

Email: sayomimatsu@yahoo.co.jp

Yoichiro Aoshima^1,2^

Email: y.aos@hama-med.ac.jp

Taisuke Akamatsu^3^

Email: redasthma@yahoo.co.jp

Yasunori Enomoto^1,2^

Email: [yasunori.enomoto@riken.jp](mailto:yasunori.enomoto@riken.jp)

Shiori Meguro^1^

Email: megu.s@hama-med.ac.jp

Isao Kosugi^1^

Email: kos180@hama-med.ac.jp

Hideya Kawasaki^1^

Email: gloria@hama-med.ac.jp

Tomoyuki Fujisawa^2^

Email: fujisawa@hama-med.ac.jp

Noriyuki Enomoto^2^

Email: norieno@hama-med.ac.jp

Yutaro Nakamura^2^

Email: [nakayuta@hama-med.ac.jp](mailto:nakayuta@hama-med.ac.jp)

Naoki Inui^4^

Email: inui@ hama-med.ac.jp

Kazuhito Funai^5^

Email: kfunai@hama-med.ac.jp

Takafumi Suda^2^

Email: suda@hama-med.ac.jp

Toshihide Iwashita^1,^*

Email: toshiiwa@hama-med.ac.jp

^1^ Department of Regenerative and Infectious Pathology, Hamamatsu University School of Medicine, 1-20-1 Handayama, Higashi-ku, Hamamatsu City, Shizuoka 431-3192, Japan

^2^ Second Division, Department of Internal Medicine, Hamamatsu University School of Medicine, 1-20-1 Handayama, Higashi-ku, Hamamatsu City, Shizuoka 431-3192, Japan

^3^ Division of Respiratory Medicine, Shizuoka General Hospital, 4-27-1 Kita Ando Aoi-ku, Shizuoka City, Shizuoka 420-8527, Japan

^4^ Department of Clinical Pharmacology and Therapeutics, Hamamatsu University School of Medicine, 1-20-1 Handayama, Higashi-ku, Hamamatsu City, Shizuoka 431-3192, Japan

^5^ First Department of Surgery, Hamamatsu University School of Medicine, 1-20-1 Handayama, Higashi-ku, Hamamatsu City, Shizuoka 431-3192, Japan

^*^ **Corresponding author**: Toshihide Iwashita, Department of Regenerative and Infectious Pathology, Hamamatsu University School of Medicine, 1-20-1 Handayama, Higashi-ku, Hamamatsu City, Shizuoka 431-3192, Japan

Telephone: +81 53 435 2223; Fax: +81 53 435 2224; E-mail: toshiiwa@hama-med.ac.jp

**Figure S1**

To quantify the number of CD248^high^ITGA8^low^ fibroblast-like cells and CD248^low^ITGA8^high^ fibroblast-like cells in IPF lungs, we first imaged serial sections stained by EVG, anti-CD248 antibody, and ITGA8 antibody and then counted the number of CD248-positive spindle cells, ITGA8-positive spindle cells, and the total number of the nuclei excluding endothelial cells. We calculated the ratio between the number of each cell type (CD248-positive spindle cells or ITGA8-positive spindle cells) and the total number of nuclei excluding endothelial cells in collagen fiber-rich connective tissue (white dotted area) and elastic fiber-rich connective tissue (white solid area), respectively.

**
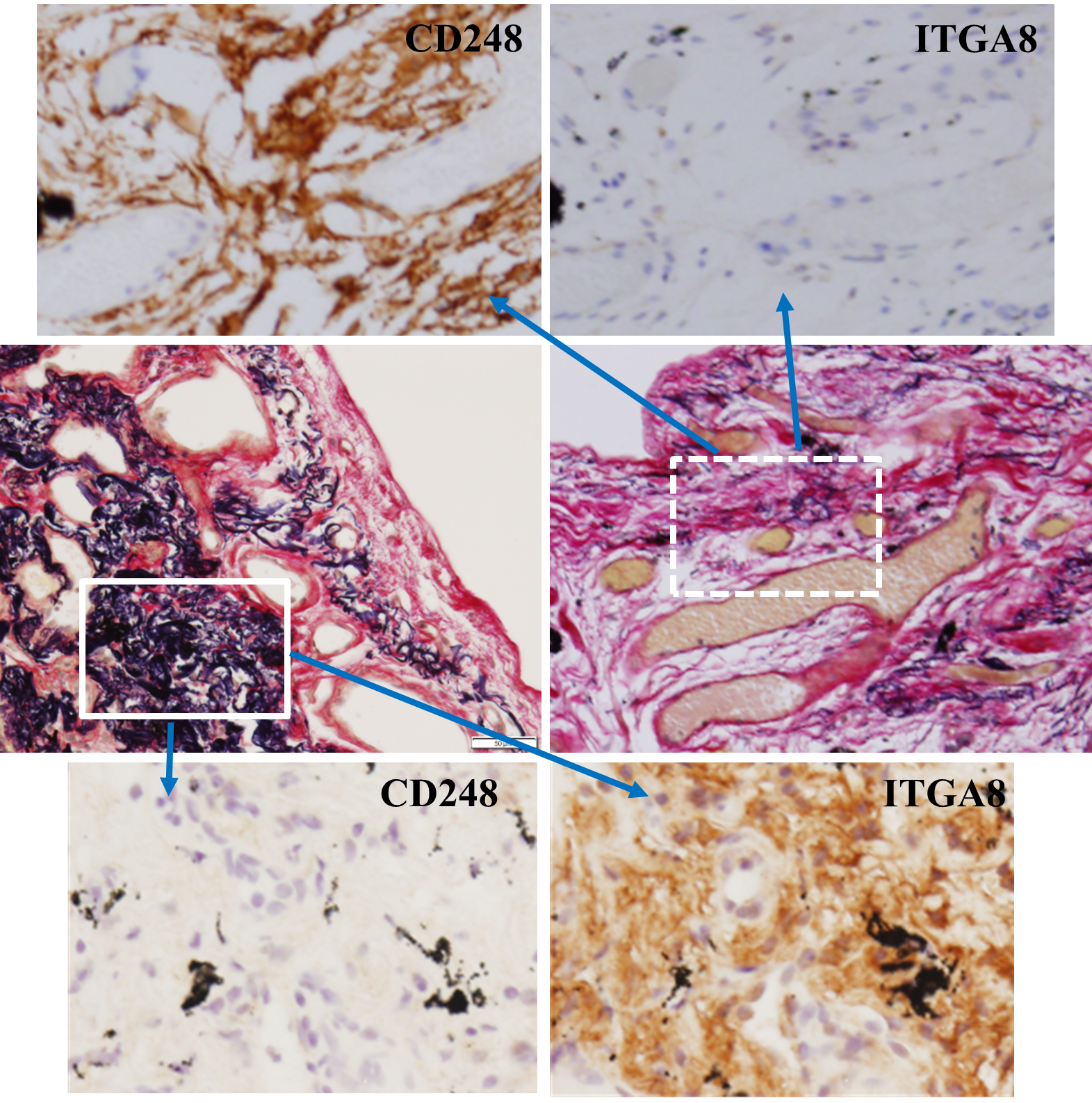
**

**Figure S2**

**
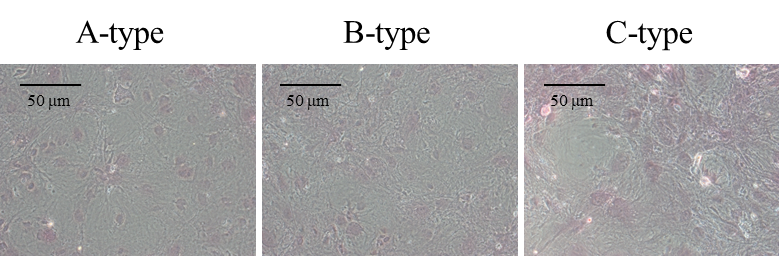
**No osteoblast differentiation was observed in any of mouse fibroblast types. Confluent cells were cultured in osteogenic differentiation medium for 21 days, and cells were stained by the von Kossa stain to detect calcium deposition. Visible calcium deposition (black) was not found in any of the fibroblast types.

**Figure S3**

**
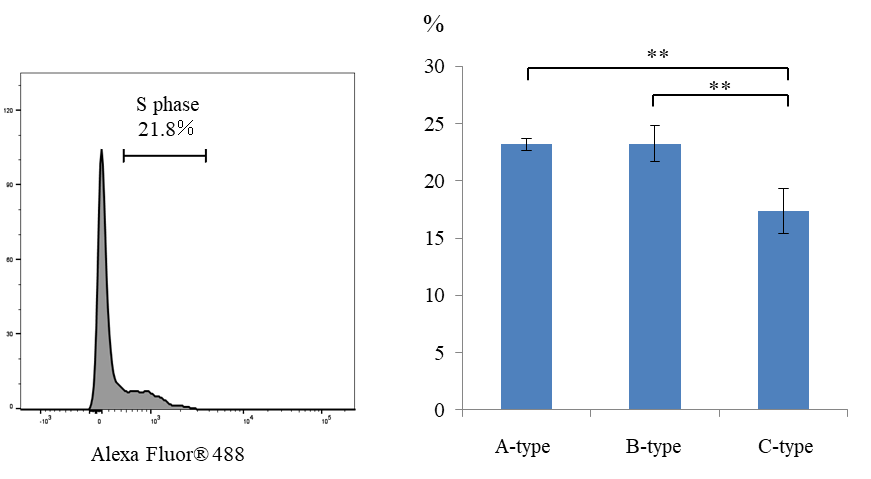
**Cell cycle of mouse fibroblast subtypes was analyzed using 5-ethynyl-2′-deoxyuridine (EdU) (Thermo Fisher Scientific), according manufacture’s instruction. *Left,* Representative example of the EdU histogram indicating cell cycle distribution. The high peak on the left side of the figure represents cells in the G_0_G_1_ phase, and the low peak on the right side of the figure represents cells in the S phase. *Right,* the proportion of each mouse fibroblast-type cells in the S phase after 4 days of culture. The experiments were performed in triplicate. Data represent mean values ± standard deviations of the results obtained in three independent experiments performed in triplicate. A total of three mice were used for this experiment. ** *P* < 0.01.

**Figure S4**

The expression levels of *Sca-1* and *Itga8* changed during A-type and C-type mouse fibroblast culture, respectively. Quantitative PCR for *Sca-1* and *Itga8* was performed using prepared cDNA samples of the freshly isolated A-type mouse fibroblasts and C-type mouse fibroblasts (Day 0) and those cultured for 7 days (Day 7), respectively. Expression was measured related to the level of glyceraldehyde-3-phosphate dehydrogenase (*Gapdh*) for each sample, which was adjusted to 100. Three independent experiments were performed in triplicates. Mean values ± standard deviations are presented. *** *P* < 0.001.


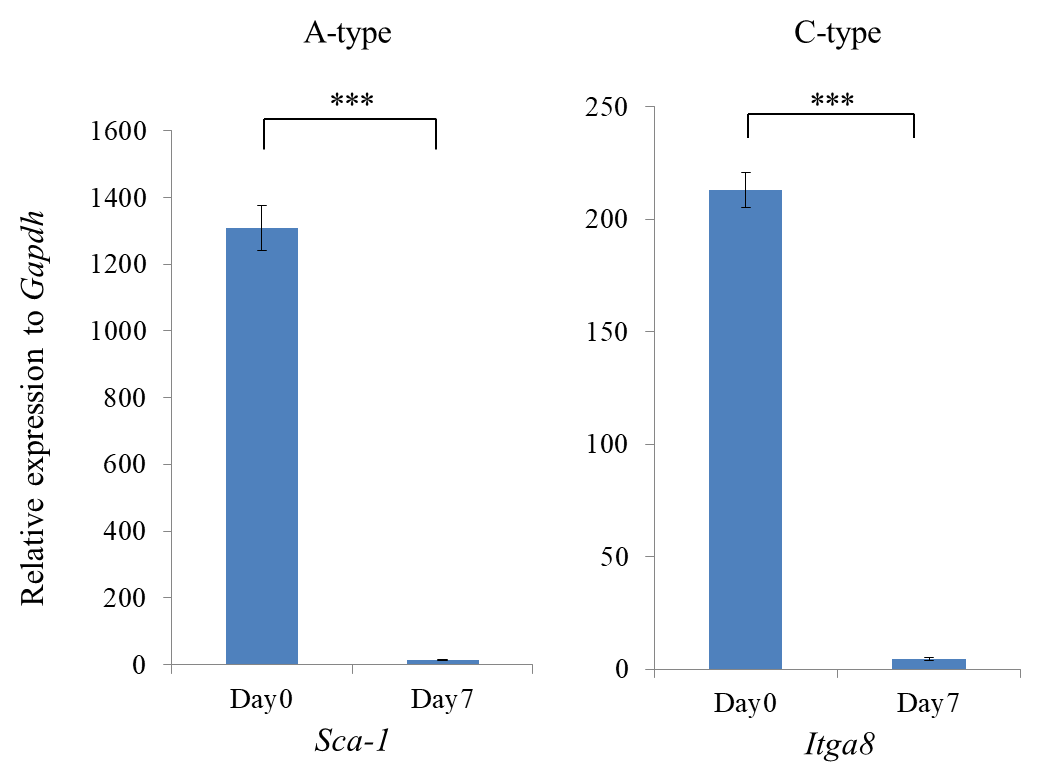


**Figure S5**

Multiple immunofluorescence (IF) images of pulmonary artery wall of normal human lung (blue, DAPI; green, lineage-specific markers; yellow, CD248; red, ITGA8). Ad indicates adventitia of pulmonary artery. Tunica indicates the smooth muscle layer of the pulmonary artery. White arrows indicate the nuclei of lineage^neg^CD248^pos^ITGA8^low^ human fibroblast-like cells. Representative results are presented in all panels; scale bars, 100 µm.


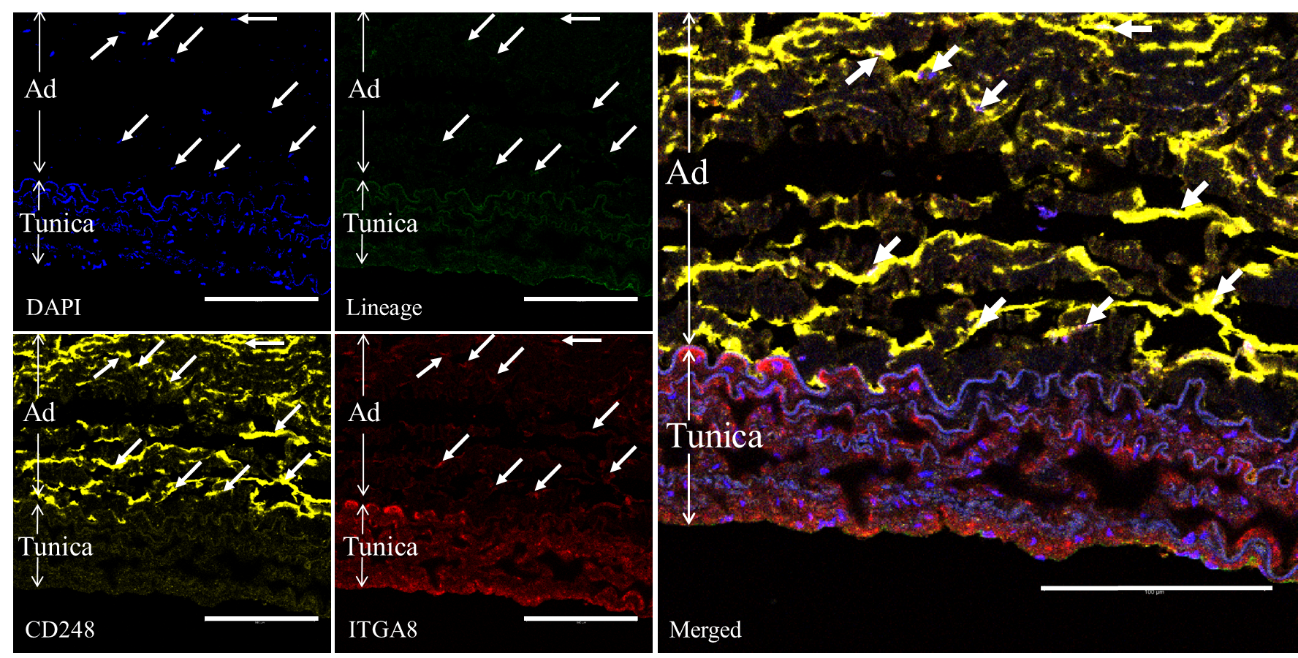


**Figure S6**


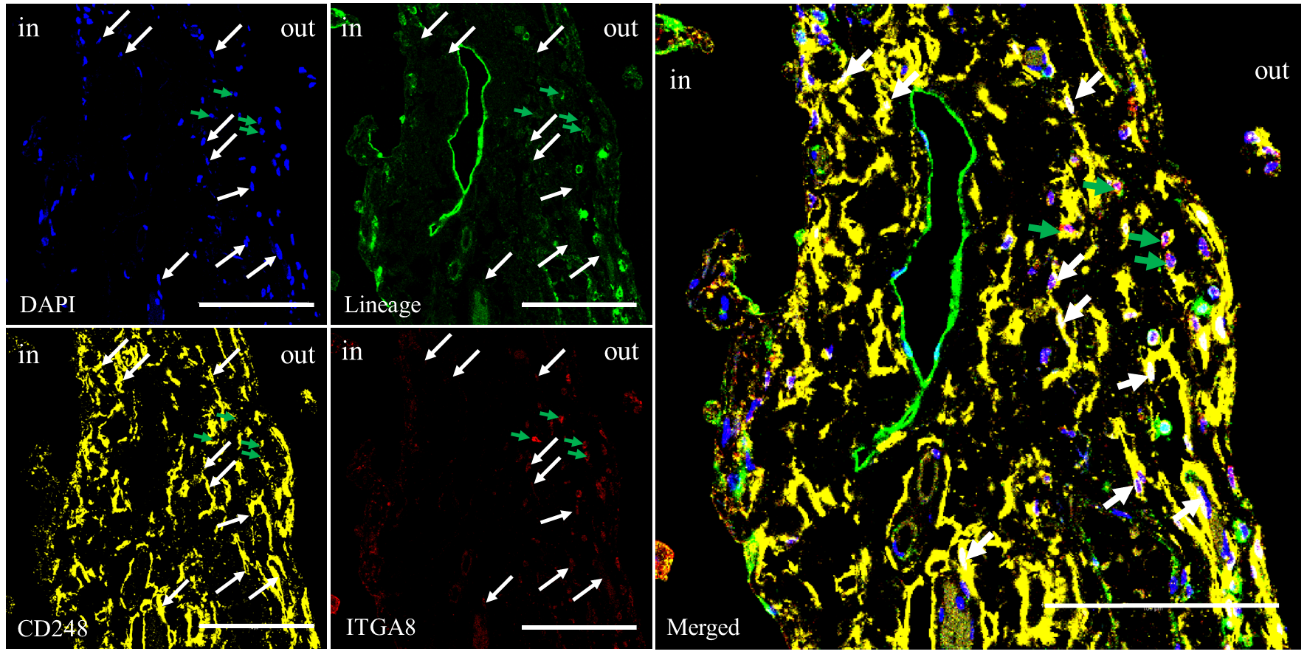
Multiple IF images of visceral pleura of normal human lung (blue, DAPI; green, lineage-specific markers; yellow, CD248; red, ITGA8). White arrows indicate lineage^neg^CD248^pos^ITGA8^low^ human fibroblast-like cells. Green arrows indicate the nuclei of lineage-positive cells with high ITGA8 expression. In and out indicate the inside and the outside of the lung, respectively. Representative results are presented in all panels; scale bars, 100 µm.

**Figure S7**

CD248-positive and ITGA8-positive human fibroblast-like cells were observed in the dermis of skin, the submucosa of large intestine, the interstitium of heart, medulla of kidney, and portal area of liver by conventional IHC (400×). Scale bars, 50 µm.


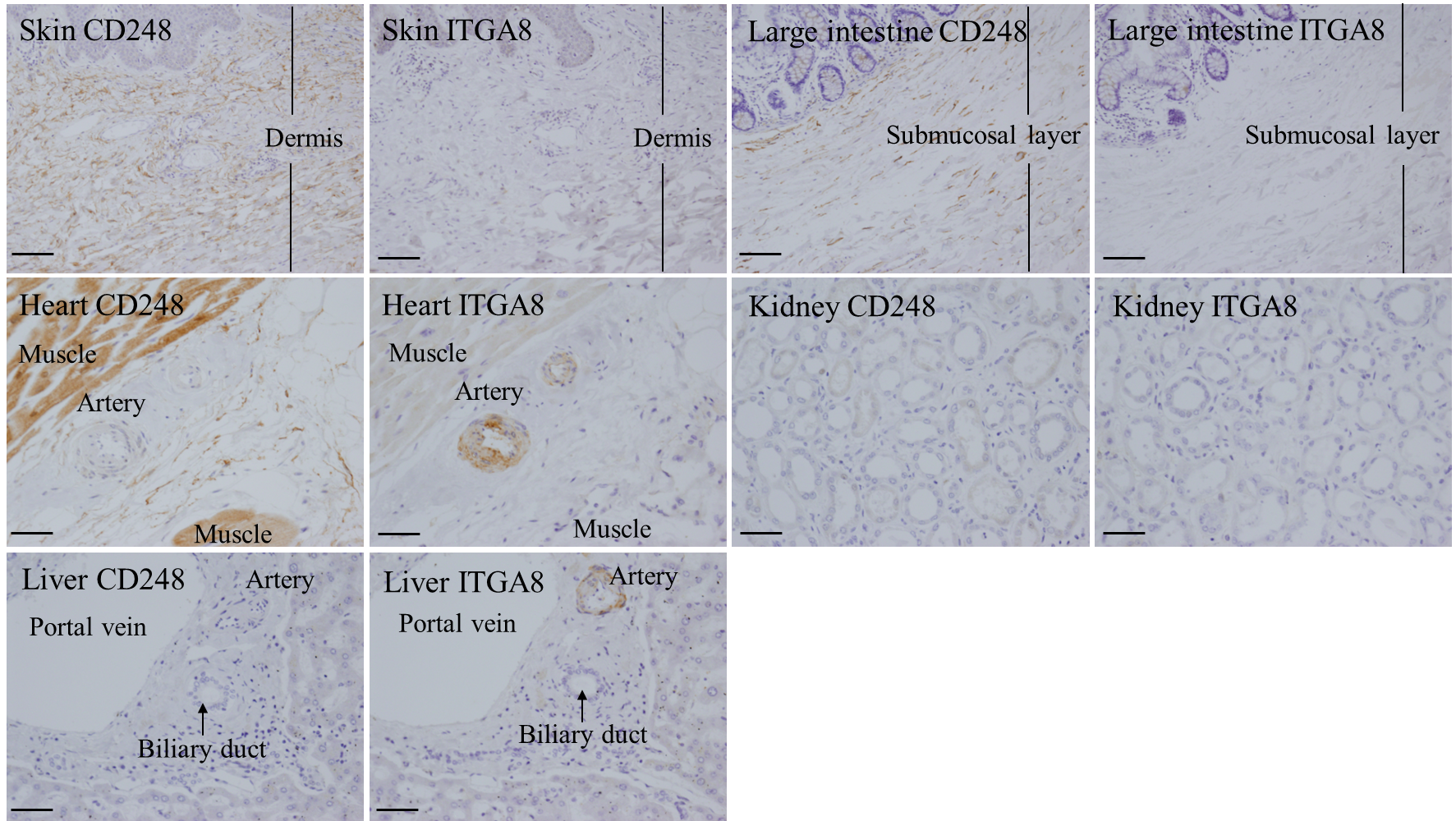


**Figure S8**

(a): We determined the relative area stained positive for CD248 by comparing the DAB-stained area with the area of nuclear staining with hematoxylin, as shown in Bartis et al [23]. Digital images taken in the idiopathic pulmonary fibrosis (IPF) and normal lung sections opened in ImageJ and DAB (brown) and hematoxylin (blue) channels were digitally separated using a plugin. After both the resulting images were converted to 8-bit grayscale, threshold levels were applied, and the resulting 1-bit image was generated. To obtain a relative CD248 staining area, we compared the number of pixels that exceeded the threshold for DAB staining and hematoxylin staining in the two images.

(b): Comparison of the relative areas stained positively for CD248 in 10 IPF lungs and 10 normal lungs. Mean values ± standard deviations are presented. *** *P* < 0.001.

**a**

**
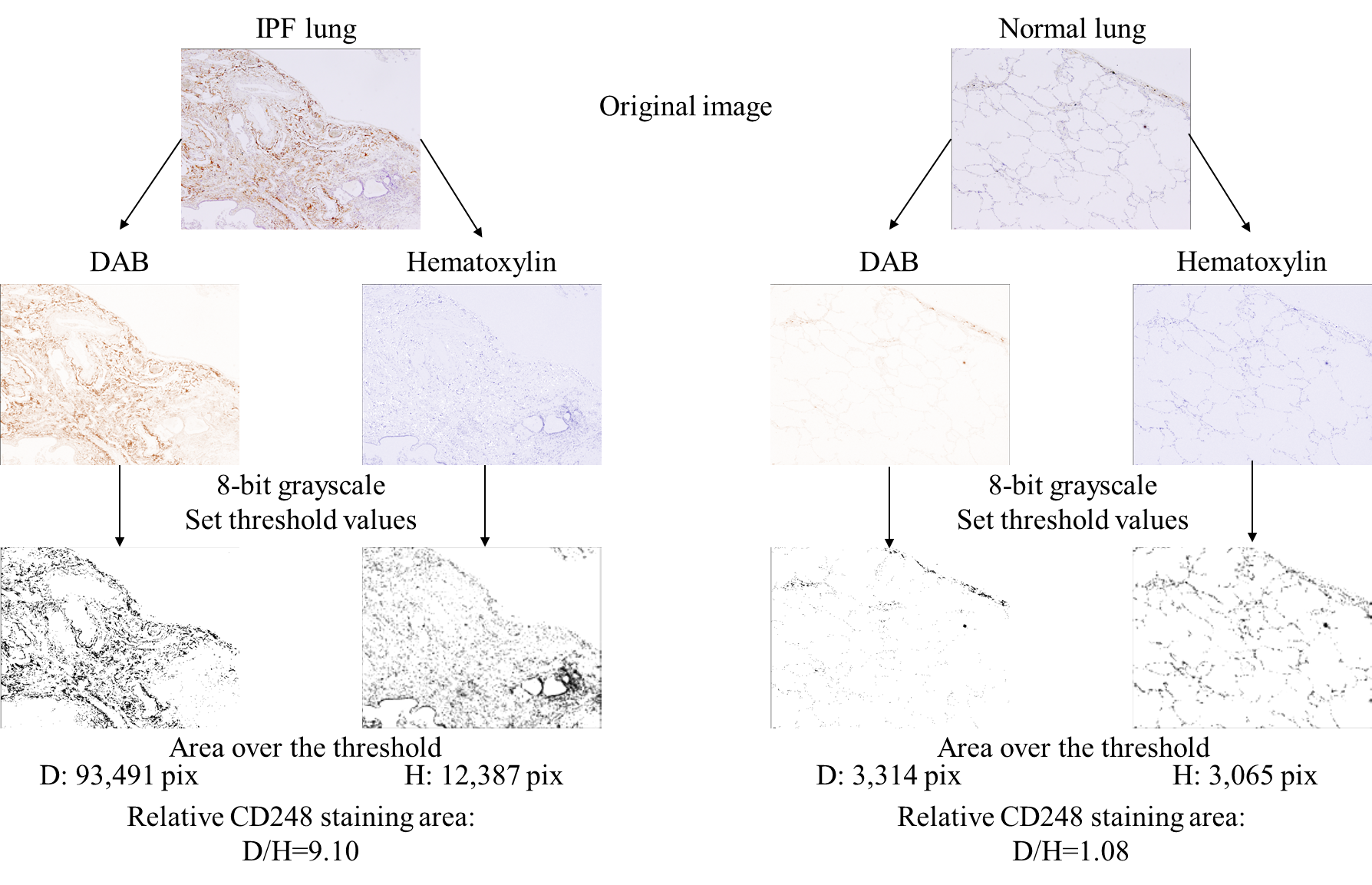
**

**b**

**
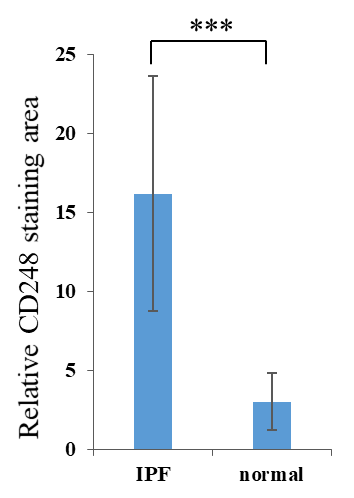
**

**Table S1**

Details of the antibodies used in this study.

**Table S2**

Microarray analysis results of pulmonary single cells (Pu) and three immunophenotypically distinct mouse fibroblast types. The Excel spreadsheet contains all data obtained in the microarray analysis of gene expression in Pu and A-, B-, and C-type fibroblasts (See attached Excel spreadsheet). GEO accession number of the microarray data is GSE102058.

**Table S3**

Specific markers associated with Sca-1^low^ mouse fibroblast (C-type fibroblast)-specific genes.

The identification strategy used for the identification of Sca-1^low^ mouse fibroblast (C-type fibroblast)-specific genes is presented here. First, 1202 genes showing an average gene expression value higher than 12.5 (log_2_-transformed value) in Sca-1^low^ mouse fibroblasts were selected. In the next step, we selected 221 of 1202 genes with average expression values less than 11.5 (log_2_-transformed value) in pulmonary single cells (Pu). In the next step, we selected 100 of 221 genes showing an average expression value that was 2.5 higher (log_2_-transformed value) in Sca-1^low^ mouse fibroblasts than those of all genes detected in pulmonary single cells (Pu). In the next step, we selected 23 of 100 genes showing an average expression value that was 0.5 higher (log_2_-transformed value) in Sca-1^low^ mouse fibroblasts than those of all genes detected in Sca-1^high^ mouse fibroblasts.

**Table S4**

Specific markers associated with Sca-1^high^ mouse fibroblast (A- and B-type fibroblast)-specific genes.

The identification strategy used for the identification of Sca-1^high^ mouse fibroblast (A- and B-type fibroblast)-specific genes is presented here. First, 1165 genes showing an average gene expression value higher than 12.5 (log_2_-transformed value) in Sca-1^high^ mouse fibroblasts were selected. In the next step, we selected 187 of 1165 genes with average expression values less than 11.5 (log_2_-transformed value) in pulmonary single cells (Pu). In the next step, we selected 91 of 187 genes showing an average expression value that was 2.5 higher (log_2_-transformed value) in Sca-1^high^ mouse fibroblasts than those of all genes detected in pulmonary single cells (Pu). In the next step, we selected 36 of 91 known genes showing an average expression value that was 0.5 higher (log_2_-transformed value) in Sca-1^high^ mouse fibroblasts than those of all genes detected in Sca-1^low^ mouse fibroblasts (C-type fibroblasts).

**Table S5**

Primers used for quantitative PCR used in this study.

**Table S6**

Patient demographics and clinical data. The demographic and clinical data of 10 patients with a histologically confirmed idiopathic pulmonary fibrosis (IPF) was analyzed. Sex, age, smoking status, body mass index, and lung function parameters including Forced Expiratory Volume in 1s (FEV1), Forced Vital Capacity (FVC), Total Lung Capacity (TLC), Diffusing capacity of the lungs for carbon monoxide (DLCO), and GAP index for IPF.
